# Dispersal of the Common Cutworm, *Spodoptera litura*, Monitored by Searchlight Trap and Relationship with Occurrence of Soybean Leaf Damage

**DOI:** 10.3390/insects11070427

**Published:** 2020-07-09

**Authors:** Akira Otuka, Masaya Matsumura, Makoto Tokuda

**Affiliations:** 1 Institute of Agricultural Machinery, National Agriculture and Food Research Organization, Tsukuba, Ibaraki 3058517, Japan; 2 Kyushu Okinawa Agricultural Research Center, National Agriculture and Food Research Organization, Koshi, Kumamoto 8611192, Japan; mmasa@affrc.go.jp; 3 Central Region Agricultural Research Center, National Agriculture and Food Research Organization, Tsukuba, Ibaraki 3058517, Japan; 4 Faculty of Agriculture, Saga University, Honjo, Saga 8408502, Japan; tokudam@cc.saga-u.ac.jp

**Keywords:** *Spodoptera litura*, dispersal, searchlight trap, soybean, monitoring

## Abstract

The common cutworm, *Spodoptera litura* Fabricius (Lepidoptera: Noctuidae) is a major pest of soybean. Pheromone traps are used to monitor male adults, but the catch peaks do not always predict leaf damage in soybean fields. Thus, there is no accurate means of forecasting soybean damage, and insecticide is applied on the basis of farmers’ observations of actual damage in fields. To understand the occurrence of soybean leaf damage, the dispersal of *S. litura* in a soybean field in southwestern Japan was preliminarily investigated using a searchlight trap in comparison to a pheromone trap at one location, from August to mid-October in 2016–2018. To determine the relationship between pest arrival and crop damage, trap catch numbers and the number of soybean leaves damaged by 1st-instar larvae were examined by separately comparing raw numbers and cumulative numbers. The raw catch numbers of the two trap types in August and September 2016 and 2018 preceded subsequent damage peaks by an average of 5.3 days. This temporal difference coincided with the estimated duration of the egg stage plus an assumed mating period. Furthermore, the cumulative catch numbers of the two traps in August and September were linearly associated with cumulative damaged leaves in the same period in each year and in the three-year period. The coefficient of determination (*R*^2^) of linear regression between the cumulative catch numbers of the searchlight trap and the cumulative damaged leaves for the three-year period was much higher than that between the cumulative catch of the pheromone trap and cumulative damage. This suggests that soybean leaf damage is closely linked to the number of *S. litura* arrivals at the survey site. Thus, the searchlight trap captured *S. litura* arrivals better than the pheromone trap. As the linear regression function of the cumulative catch of the searchlight trap for the three-year period was tentatively correlated with a prefectural economic injury level for soybean fields, it might be feasible to predict *S. litura*-induced soybean damage using searchlight traps. The cumulative female catch number of the searchlight trap was also linearly associated with damaged leaves, but the coefficient of determination was generally lower than that with the cumulative total catch. The female ratio of searchlight trap catches in September was <0.5 in contrast to *S. litura* migrating overseas (>0.5). The advantages and disadvantages of the two trapping methods, as well as necessary further studies are discussed. Our findings provide a foundation for *S. litura* monitoring with searchlight traps.

## 1. Introduction

The common cutworm, *Spodoptera litura* Fabricius (Lepidoptera: Noctuidae) is a polyphagous insect pest that attacks agricultural crops such as soybean, maize, vegetables, and fruit trees [1]. A sex pheromone of *S. litura* was first identified and synthesized in the early 1970s [2] and is frequently used to monitor male adults in fields. Previous studies that investigated the relationship between the male catch of pheromone traps, using a pheromone lure or a virgin female adult, and the number of moth egg masses laid on the leaves of taro (*Colocasia esculenta* [L.] Schott) and groundnut (*Arachis hypogaea* L.), showed that male catch peaks occur before, during, or after peaks in egg mass number [3,4,5]. Similar results have been reported in soybean [6].

Soybean is a major summer crop cultivated in southwestern Japan and is typically attacked by *S. litura* larvae from mid-August to September. The 1st- and 2nd-instar larvae eat only soft mesophyll tissues and leave the leaf epidermis, which results in an obvious pattern of fine white dots on green leaves in fields. One study investigated the relationship between the male catch of a pheromone trap and the number of leaves newly damaged by 1st-instar larvae from July to October over four years; the results revealed that the catch peaks mainly coincided with the number of newly damaged leaves, but varied by ±3 days [6]. In other words, the catch peak occurred before or *after* leaf damage. Therefore, the factors that underlie the temporal changes in pheromone trap catches appear to be complex. The notion that the male adult catch increases before damage occurs does not always fit observations; the trap catch can increase even after the leaf damage has occurred in some cases. The soybean study mentioned above also demonstrates the difficultly of using early trap peaks in August and subsequent temperature trends to forecast the bulk of damage occurring in September [6]. Thus, there is no accurate means of forecasting soybean damage, and whether additional application of insecticide is needed is currently determined on the basis of farmers’ observation of actual damage in fields.

*Spodoptera litura* actively disperse in windy weather [7] and might even migrate long distances overseas to Japan [8]. Thus, their dispersal from other areas into target fields requires further study [6]. Furthermore, while monitoring females is essential (because they lay eggs), pheromone traps only capture males.

A searchlight trap with a narrow and strong visible light beam aimed vertically was developed by the Institute of Plant Protection, Chinese Academy of Agricultural Sciences [9] and it can capture various migratory insects [9,10,11,12,13,14,15,16,17,18,19], including both sexes of *S. litura* [20]. Traditional pheromone traps capture males only, but females produce subsequent generations, which attack soybean. Therefore, searchlight traps could be used to monitor the dispersal of both female and male *S. litura*.

Hence, this study investigated the dispersal of *S. litura* by using both a searchlight trap and a pheromone trap to determine the relationship between the arrival of *S. litura* and soybean leaf damage.

## 2. Materials and Methods

### 2.1. Survey Site and Trap Monitoring

**Figure 1:**
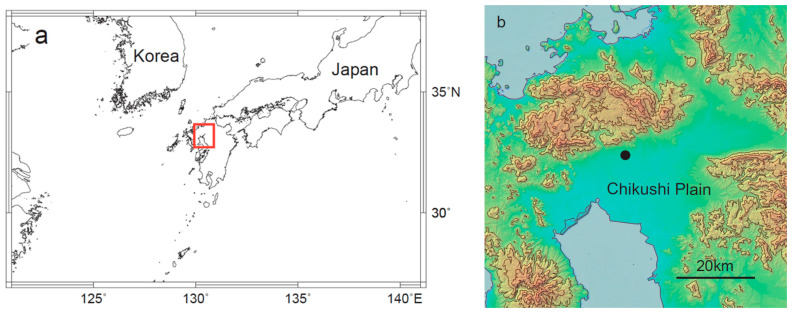
Study area. (**a**) Study area location and (**b**) zoomed-in contour map (100-m intervals) of the area outlined in (**a**). The black dot in (**b**) indicates the monitoring site (the contour map was generated by Web Contour Maker of Japan: http://ktgis.net/service/webcontour/index.html [21]).

The survey was conducted in the flat Chikushi Plain in southwestern Japan (Figure 1a). The plain is bounded by an inland sea to the south and mountains (maximum elevation: ~1000 m) to the other 3 directions (Figure 1b). Soybean and rice are the main summer crops in the plain, and their fields are rotated annually.

*Spodoptera litura* occurrence and soybean damage due to larvae were surveyed from 2016–2018. A searchlight trap and a pheromone trap were set up at a fixed monitoring site for the 3-year study period (33.305° N, 130.329° E; black dot in Figure 1b). The searchlight trap emits a bright vertical visible light beam. It has 4 major components: a 1000-watt metal halide lamp (M1000B/BD, Iwasaki Electric Co., Ltd., Tokyo, Japan), lamp case with a parabolic reflector (H567SX), metal funnel 1.2 m in diameter, and insect collection box placed under the funnel [9,19]. A vinyl chloride resin board impregnated with dichlorvos insecticide (*Bapona*; Earth Corporation, Tokyo, Japan) was placed inside the box to kill caught insects. The searchlight trap was automatically switched on at 1900 h and off at 2400 h local time from 12 August to 14 October. This trapping period was selected to match the active flight hours of nocturnal *S. litura* moths. Trapped samples were transferred from the collection box to a plastic bag, which was stored at −20 °C the next morning.

The species and sex of *S. litura* were identified by examination of genital morphology under a stereomicroscope. Briefly, genitalia were extracted from the abdomen, dissolved in 0.5-N KOH solution, and heated in an oven at 80 °C for 1 h. Samples wet by rain in 2016 were excluded because of poor sampling quality. However, all daily samples in 2017 and 2018 were included, because the collection box of the trap was modified to ensure that the rainwater quickly drained from the box. No special permission was needed for this survey, because the site is not located in any protected area and no protected species were sampled.

Meanwhile, a pheromone funnel trap suspended on a pole 1.2 m above ground was set 60 m east of the searchlight trap during the 3-year survey. Samples were collected and counted every morning. A pheromone lure—an SE lure impregnated with a 5-mg mixture of (Z,E)-9,11-tetradecadienyl acetate and (Z,E)-9,12-tetradecadienyl acetate (10:1; Sankei Chemical Co., Ltd., Kagoshima, Japan)—was changed monthly. Monitoring was not conducted on several days, because of windy weather conditions.

### 2.2. Damage Survey in Soybean Fields

A popular regional variety of soybean, ‘*Fukuyutaka*’, was seeded in late July every year in a 30-a (3000 m^2^) experimental field in 2016 and 2018 or 50-a (5000 m^2^) experimental field in 2017. The monitored fields were changed owing to crop rotation of the area. The survey fields were located 300 m west of the traps in both 2016 and 2018, and 550 m north of the traps in 2017. The previous crop in each field was rice. Conventional cultivation management was applied, except no insecticide was used. The occurrence of damage due to *S. litura* on all leaves of 1000 soybean plants in each survey field was investigated every Monday, Wednesday, and Friday from mid-August to mid-October [6]. From mid-September, only 500 soybean plants were surveyed, because the number of leaves per plant became rather large; these numbers of leaves were selected on the basis of a previous report [6], which precisely indicated the occurrence of soybean damage in the monitored field. The survey area was approximately 10 a and contained 1000 plants seeded using a conventional method.

For comparison, the economic injury level set by Japanese prefectures is generally 5 damaged spots with whitish leaves per 1 a (100 m^2^). Herein, “spot” refers to a single small area containing at least 1 damaged plant with whitish leaves. In commercial production, farmers and agricultural companies first investigate damage by observing soybean plants from outside their fields. Then, they scrutinize damaged plants in the fields when the damage looks severe. They also use occurrence information issued by the local plant protection station, which monitors the occurrence of insects and diseases by conducting daily pheromone trap surveys and field surveys of 22 fields in the prefecture twice a month from early August to early October.

Leaf damage was assessed by counting the number of leaves that had new fine white food mark dots and green hatchlings of *S. litura* on the lower surface. To facilitate easy comparisons of trap catch numbers and numbers of damaged leaves in trend graphs, the numbers of newly damaged leaves per 1000 or 500 plants were multiplied by a factor of 5 or 10, respectively, in order to set a base number of 5000 plants. For statistical analysis (see Section 2.3), a base number of 1000 plants was used. Therefore, the number of newly damaged leaves per 500 plants in September was multiplied by a factor of 2.

Although increasing the number of replicates at the survey site was preferable for study reliability, only 1 of each type of trap and 1 monitoring field were used for two reasons. First, *S. litura* mostly occurs synchronously on the plain, which is a relatively narrow area extending 30 km north–south and 60 km east–west. Another pheromone trap is operated by the local plant protection station 10 km south of the survey site. Its catches showed similar occurrence trends (data not shown), indicating that differences in *S. litura* occurrence are relatively small within the plain; this was corroborated by observations of actual damage in fields in this region. The second reason is costs. The soybean survey was particularly expensive, and funds for such initial basic research are limited. Therefore, we conducted a single-site, 3-year study as a pilot study and discuss future research to obtain more reliable and applicable results.

### 2.3. Statistical Analysis

The correlation between the total catch number of the searchlight trap (daily) and the number of newly damaged leaves per 1000 plants (at 2- or 3-day intervals) was tested using a *date shift* method and a *cumulative* method.

In the *date shift* method, the catch data—the total and female catch numbers of the searchlight trap, and the male catch number of the pheromone trap—were shifted by +3 to +6 days against the damaged leaves data. The range approximately corresponds to the egg stage of *S. litura* (i.e., 3.2–4.3 days at an air temperature of 25 °C [22,23]) plus 2 days. The data pairs for correlation analysis were then automatically generated by selecting both values on the same date in each dataset. The duration was set from 12 August to 30 September, because *S. litura* arrival mainly occurs in late August and late September and there were very few damaged leaves from early October (below). This might be because of the degradation of plants preferred as host plants by the insects. The number of analyzed data pairs using this method was less than or equal to the sample number of the damaged leaf survey (i.e., the number of survey dates at 2- or 3-day intervals) owing to missing trap data due to heavy rain or typhoons.

In the *cumulative* method, correlations were determined using the cumulative trap catches, total or female catches, and numbers of damaged leaves. Daily cumulative values for each trap were calculated by repeatedly adding the preceding cumulative catch to the current catch from 12 August to 30 September. When no catch was recorded due to rain in 2016, a value of 0 was assumed for that date because the catches of searchlight traps are generally very small when it rains, based on the surveys in the later years. To calculate the cumulative numbers of damaged leaves, only values from soybean survey dates (i.e., every Monday, Wednesday, and Friday) were calculated by adding the preceding cumulative value to the current one. Hence, the cumulative values were obtained at 2- to 3-day intervals. Then, the cumulative trap catch of each trap type was shifted by +3 to +6 days against the cumulative damage number in order to determine how the date shift affected the correlations. Accordingly, paired cumulative trap catches and numbers of damaged leaves on the soybean survey dates were plotted on a scatter plot, and linear regression analysis was performed. In addition, the cumulative trap catches of both trap types were directly plotted in a scatter plot, followed by a linear regression analysis.

The correlations between the catch numbers of the searchlight trap and numbers of newly damaged leaves determined by both methods were calculated by the Spearman’s rank correlation test using the *cor.test* function in R version 3.6.1 (https://www.r-project.org/). The catch number per 500 plants since mid-September was multiplied by 2 to obtain the catch number per 1000 plants. The correlations between male catch numbers in the pheromone trap and the numbers of newly damaged leaves were determined in the same manner. Linear regression analysis was subsequently performed using the *lm* function in R, yielding coefficients of determination (*R*^2^).

Given that the searchlight trap captures adults of both sexes [20], the ratio of females in the trap catches and seasonal variations were determined from mid-August to mid-October and compared with data from the literature. The ratio was calculated daily as the ratio of the female catch number to the total catch number. To obtain reliable ratios, only values calculated when the total catch exceeded 10 catches/night were used.

## 3. Results

### 3.1. Searchlight Trap Catch and Soybean Damage Trends

**Figure 2:**
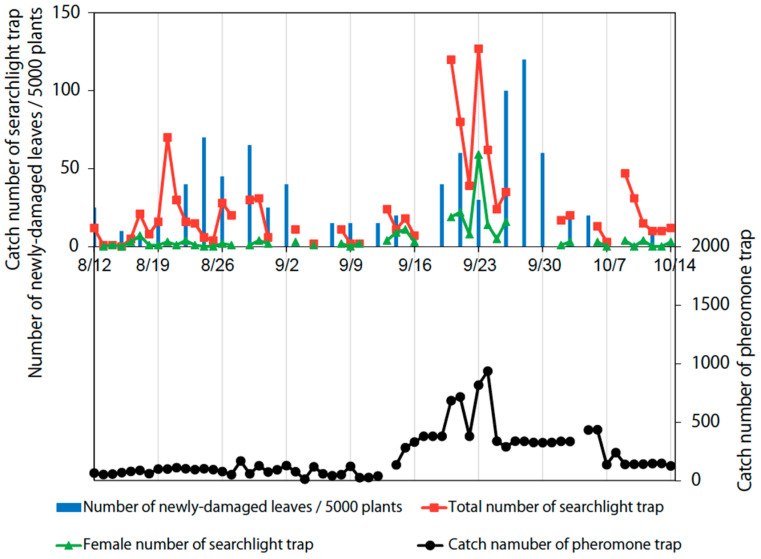
Temporal variation of *S. litura* catch numbers caught by the searchlight and pheromone traps, and numbers of newly damaged leaves per 5000 soybean plants in 2016 (Saga, western Japan).

**Figure 3:**
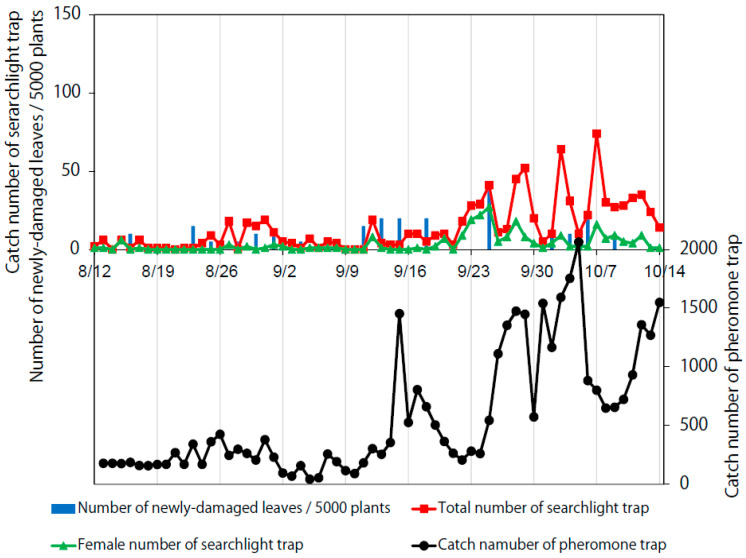
Temporal variation of *S. litura* catch numbers caught by the searchlight and pheromone traps, and numbers of newly damaged leaves per 5000 soybean plants in 2017.

**Figure 4:**
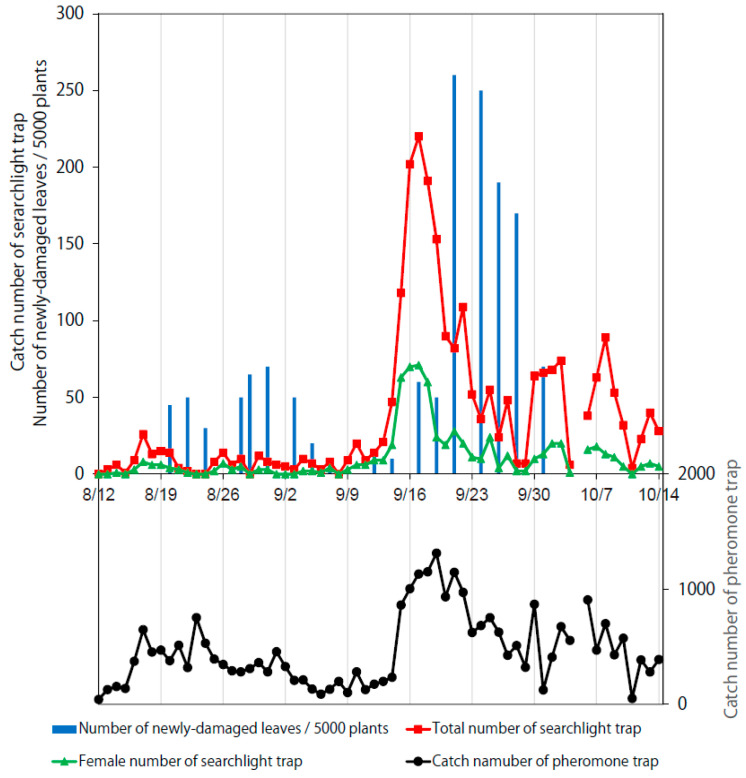
Temporal variation of *S. litura* catch numbers caught by the searchlight and pheromone traps, and the numbers of newly damaged leaves per 5000 soybean plants in 2018.

The catch number of the searchlight trap and the numbers of newly damaged leaves varied by year: they were largest in 2018, followed by 2016 and 2017 (Figure 2, Figure 3 and Figure 4). Large total catch number peaks of the searchlight trap in 2016 and 2019 (red lines) appeared a few days before peaks of the number of newly damaged leaves (blue bars).

### 3.2. Correlations Calculated Using the Date Shift Method

**Figure 5:**
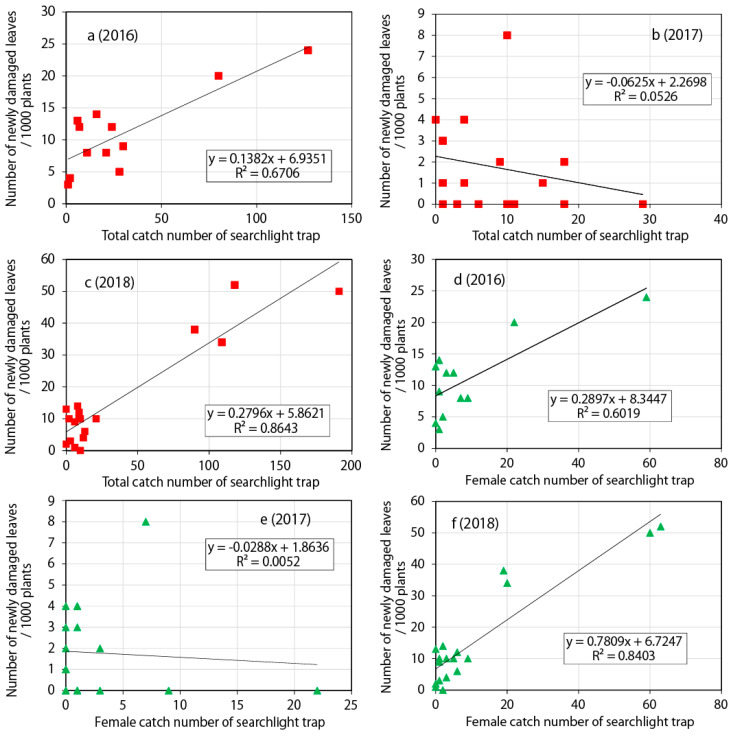
Linearity between the catch number of the searchlight trap and the number of newly damaged leaves in August and September 2016–2018. Total catch numbers of the searchlight trap in (**a**) 2016 (date shift *ds* = 5, *n* = 12), (**b**) 2017 (*ds* = 5, *n* = 19), and (**c**) 2018 (*ds* = 6, *n* = 18). Female catch number of the searchlight trap in (**d**) 2016 (*ds* = 5, *n* = 12), (**e**) 2017 (*ds* = 5, *n* = 19), and (**f**) 2018 (*ds* = 6, *n* = 18).

Correlations were calculated using the date shift method. The total catch numbers of the searchlight trap were significantly correlated with the numbers of newly damaged leaves in August and September in 2018 (Spearman’s rank correlation test: date shift [*ds*] = 6, *ρ* = 0.51, *P* = 0.029), but not 2016 (*ds* = 5 days, *ρ* = 0.57, *P* = 0.054) and 2017 (*ds* = 5, *ρ* = −0.32, *P* = 0.182). However, a weak linearity is visible on a scatter plot of the 2016 case (Figure 5a). Similarly, the female catch numbers in the searchlight trap were significantly correlated with the numbers of newly damaged leaves in 2018 (*ds* = 6, *ρ* = 0.67, *P* < 0.01), but not 2016 (*ds* = 5, *ρ* = 0.414, *P* = 0.181) and 2017 (*ds* = 5, *ρ* = −0.088, *P* = 0.721).

In linear regression analysis, the coefficient of determination peaked at *ds* = 5 and 6 in 2016 and 2018, respectively (Table 1). Furthermore, the total catch number of the searchlight trap exhibited a slightly stronger linearity than the female catch number (coefficients of determination of the total and female catches: 0.6706 and 0.6019 in 2016, respectively; 0.8643 and 0.8403 in 2018, respectively) (Table 1, Figure 5).

### 3.3. Comparison of the Searchlight and Pheromone Traps According to the Date Shift Method

**Figure 6:**
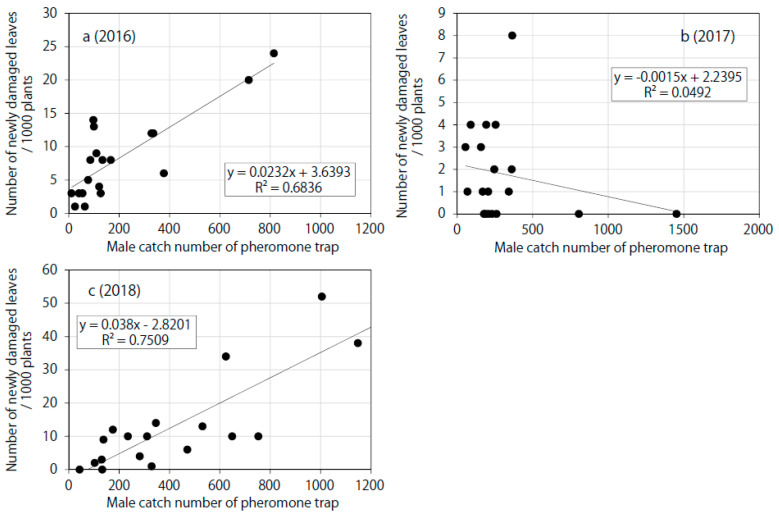
Linearity between the male catch number of the pheromone trap and the number of newly damaged leaves in August and September in (**a**) 2016 (*ds* = 5, *n* = 19), (**b**) 2017 (*ds* = 5, *n* = 17), and (**c**) 2018 (*ds* = 5, *n* = 18).

The male catch numbers of the pheromone trap were significantly correlated with the numbers of newly damaged leaves in August and September 2016 (Spearman’s rank correlation test: *ds* = 5, *ρ* = 0.70, *P* < 0.001) and 2018 (*ds* = 5, *ρ* = 0.79, *P* < 1 × 10^−4^ ) but not 2017 (*ds* = 5, *ρ* = −0.20, *P* = 0.405). The coefficient of determination for the pheromone trap peaked at *ds* = 5 (Table 2, Figure 6). The overall frequency of maximum coefficients of determination at *ds* = 5 and 6 for the traps in 2016 and 2018 were 3 and 1, respectively (see total catch and male catch in Table 1). Hence, the mean date shift was 5.3 days. The coefficients of determination for the pheromone trap in 2016 (*R*^2^ = 0.6836) and 2018 (*R*^2^ = 0.7509) are close to or less than those for the total catch number of the searchlight trap (*R*^2^ = 0.6706 and 0.8643, respectively). The daily total catches of the searchlight trap and the pheromone trap exhibited significant correlations for all 3 years (*ρ* = 0.57, *P* < 0.001, *R*^2^ = 0.64 in 2016; *ρ* = 0.52, *P* < 0.001, *R*^2^ = 0.28 in 2017; *ρ* = 0.64, *P* < 1 × 10^−6^, *R*^2^ = 0.67 in 2018).

### 3.4. Comparison of the Searchlight and Pheromone Traps According to the Cumulative Method

**Figure 7:**
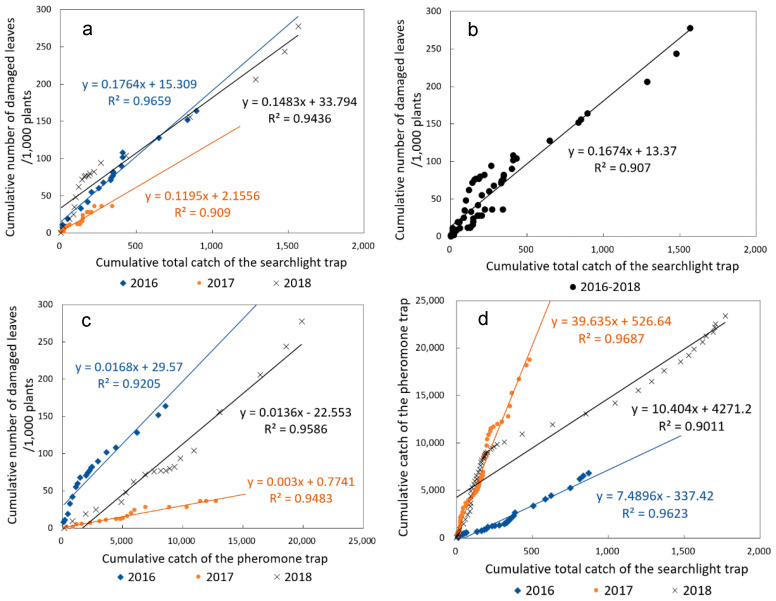
Linear regression analysis of cumulative total catch numbers and numbers of damaged leaves. (**a**) Linearity between the cumulative total catch number of the searchlight trap and the cumulative number of damaged leaves at *ds* = 4 for each year. (**b**) Linearity between the cumulative total catch number of the searchlight trap and the cumulative number of damaged leaves at *ds* = 4 for 2016–2018. (**c**) Linearity between the cumulative catch number of the pheromone trap and the cumulative number of damaged leaves at *ds* = 4. (**d**) Linearity between the cumulative catch numbers of the pheromone trap and the searchlight trap in August and September.

The cumulative total catch of the searchlight trap and the cumulative number of newly damaged leaves exhibited a correlation (Spearman’s rank correlation tests: *ds* = 4, *ρ* = 0.999, *P* < 1 × 10^−15^ in 2016; *ds* = 4, *ρ* = 0.994, *P* < 1 × 10^−15^ in 2017; *ds* = 4, *ρ* = 0.999, *P* < 1 × 10^−15^ in 2018; Figure 7a). All coefficients of determination exceeded 0.90 (Table 2) and were greater than those calculated using the previous method. The slopes of linear regression ranged from 0.1195 to 0.1764 at *ds* = 4, which were close to each other (Figure 7a). During the 3-year study period, the cumulative total catch of the searchlight trap and the cumulative number of the damaged leaves also exhibited a correlation (*ds* = 4, *ρ* = 0.916, *P* < 1 × 10^−15^); the linear regression function (*y* = 0.1674*x* + 13.3697; *R*^2^ = 0.9070) was obtained (Figure 7b).

Furthermore, the cumulative female catch of the searchlight trap and the cumulative number of the damaged leaves exhibited a correlation (*ds* = 6, *ρ* = 0.999, *P* < 1 × 10^−15^ in 2016; *ds* = 6, *ρ* = 0.987, *P* < 1 × 10^−14^ in 2017; *ds* = 6, *ρ* = 0.999, *P* < 1 × 10^−15^ in 2018). However, most coefficients of determination were smaller than those of the cumulative total catch of the searchlight trap (Table 2).

The cumulative catch of the pheromone trap and the cumulative number of the damaged leaves also exhibited a correlation (*ds* = 4, *ρ* = 1, *P* < 1 × 10^−5^ in 2016; *ds* = 4, *ρ* = 0.995, *P* < 1 × 10^−15^ in 2017; *ds* = 4, *ρ* = 0.999, *P* < 1 × 10^−15^ in 2018; Figure 7c); all coefficients of determination exceeded 0.92. However, the 3 regression lines were separate from each other, and the linear regression slope in 2017 was smaller than those in 2016 and 2018. In addition, the cumulative catch of the pheromone trap and the cumulative number of damaged leaves exhibited a correlation for the 3-year study period (*ds* = 4, *ρ* = 0.59, *P* < 1 × 10^−5^), and the linear regression function (*y* = 0.00879*x* + 11.7559) was obtained (Figure 7c); however, the coefficient of determination was 0.4894, which is smaller than that of the searchlight trap.

The cumulative total catch of the searchlight trap and the cumulative catch of the pheromone trap also exhibited linearity for each year (*ρ* = 0.999, *P* < 1 × 10^−15^ in 2016; *ρ* = 0.999, *P* < 1 × 10^−15^ in 2017; *ρ* = 0.999, *P* < 1 × 10^−15^ in 2018; Figure 7d); the coefficients of determination all exceeded 0.9. However, the 3 regression lines were separate, and 2 different slopes were evident: one for 2016 and another for 2017 (Figure 7d). Moreover, the linear regression for 2018 exhibited 2 parts with different slopes: when the cumulative total catch of the searchlight was less than 250, the slope was close to that in 2017, whereas the slope beyond the point was close to that in 2016 (Figure 7d).

### 3.5. Sex Ratio in the Searchlight Trap

**Figure 8:**
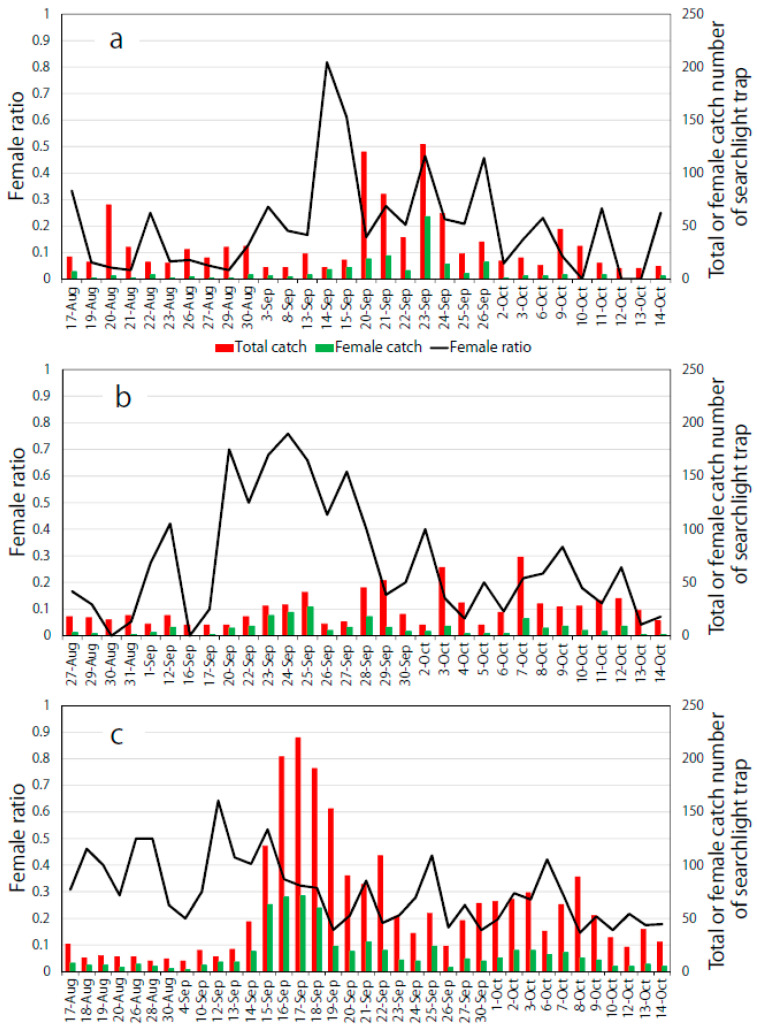
Ratio of *S. litura* females caught by the searchlight trap in (**a**) 2016, (**b**) 2017, and (**c**) 2018 in Saga Prefecture, Japan.

The ratio of females caught was less than 0.5 at most observation dates, whereas ratios exceeding 0.5 only occurred in mid-to-late September (Figure 8). The ratios of cumulative females to the total catches in 2016, 2017, and 2018 were 0.215, 0.264, and 0.289, respectively.

## 4. Discussion

The present study revealed that the catches of both the searchlight and pheromone traps were associated with soybean leaf damage according to the two evaluation approaches. The male catch numbers of the pheromone trap were significantly correlated with the numbers of damaged leaves in 2016 and 2018, but not and 2017 when the occurrence of both *S. litura* and soybean leaf damage were very small. The total and female catch numbers of the searchlight trap were significantly correlated with the numbers of damaged leaves in 2018, but not 2016 and 2017. In 2016 and 2018, the mean date shift was 5.3 days in the linear regression analysis (Table 1), which corresponds to the estimated duration of the egg stage (i.e., 3.2–4.3 days) at an air temperature of 25 °C [22,23]; the 1–2-day difference between these values might reflect the time taken for mating or an extended period for laying eggs. On the other hand, the coefficients of determination for the cumulative values peaked at *ds* = +3 to +6 and were relatively insensitive to the *ds* value (Table 2). This might reflect the increasing cumulative values, i.e., changes were relatively smaller against the base values.

Furthermore, the present study is the first to demonstrate that the relationship between cumulative *S. litura* catch numbers and soybean leaf damage is much stronger than that between the raw catch numbers of either type of trap and soybean leaf damage or that between the catch numbers between trap types. Such strong relationships were observed even in 2017 when *S. litura* occurrence was very small. These results suggest that soybean leaf damage in August and September is correlated with the total number of *S. litura* moth arrivals.

As an example used in some Japanese prefectures, the cumulative total catch number of the searchlight trap can be correlated (through a linear regression function) to the economic injury level of 5 damaged spots per 1 a. A “spot” is a place where there is damaged plant(s) with several damaged leaves. For subsequent evaluations, this “spot” is simply referred to as a “plant”. Hence, the economic injury level is then considered to be 5 plants/1 a. For example, given a linear regression function of *y* = 0.1674*x* + 13.37 (*ds* = 5) (Figure 7b), a cumulative searchlight trap catch of 100 individuals (*x*) might result in 33.3 cumulative damaged leaves/1000 soybean plants (*y*). This could be interpreted as a cumulative injury level of 3.3 plants/1 a, because 1000 plants occupy approximately 10 a. Therefore, a cumulative searchlight trap catch of 219 individuals approximately corresponds to the economic injury level in this example case. As two damage peaks in August and September are expected in a typical cultivation season (Figure 2 and Figure 4), two starting points for calculating the cumulative catch should be set on the first day of each month. Thus, the economic injury level for each month can be determined. It should be noted that the prediction accuracy is relatively low when the cumulative catch is less than 500 (Figure 7b), because values can slightly diverge from the linear regression function. However, the reason for this is unclear, necessitating further investigation of early leaf damage.

The monitoring and damage parameters determined thus far suggest that the searchlight trap is sufficiently sensitive to capture leaf damage levels in fields. Hence, it might be possible to utilize searchlight traps to predict soybean damage in the short term, although additional field studies are required to confirm the prediction accuracy and effectiveness of controlling the moth larvae. Moreover, the searchlight trap catch peaks in mid- or late August preceded the larger catch peaks in mid- or late September by one month; this phenomenon was clear in 2016 and 2018 (Figure 2 and Figure 4) and even visible in 2017 (see small increases of the red plot from 25 August–1 September and large peaks in late September in Figure 3). This suggests the possibility of predicting the timing of *S. litura* arrival in the following month. Thus, these results collectively provide a new basis for monitoring *S. litura* using searchlight traps.

While, the cumulative catch number of the pheromone trap appears complex: the linear relationship between the cumulative catch number of the pheromone trap and the cumulative number of damaged leaves changed substantially year to year (Figure 7c), resulting in a low overall coefficient of determination for the 3-year study period (Section 3.4). Therefore, it is difficult to predict the cumulative leaf damage based on the cumulative catch of pheromone traps because of the relatively large prediction error. Hence, the catches of both types of traps were linearly correlated with leaf damage, indicating that both types of traps are effective for monitoring *S. litura*. However, the pheromone trap catches did not exhibit consistent linearity throughout the study period. Thus, the temporal changes in the pheromone trap catch appear to be more complex than the changes in the searchlight trap catch.

Regarding the relationship between the cumulative catches of the two types of traps (Figure 7d), the slopes of the linear regression functions differed between 2016 and 2017. In 2017, when *S. litura* occurrence was small, the catch of the pheromone trap increased as much as 5.3 times faster than that in 2016, against the catch of the searchlight trap. Although the reason for this is unknown, this result suggests that the dispersal of *S. litura* at higher altitudes as well as male adults’ dispersal near the ground might vary and that these dispersal modes might occur with variable relative intensity. If so, dispersal at higher altitudes mainly occurred in 2016, whereas male adult dispersal near the ground mostly contributed to the pheromone trap catches in 2017. Of note, in the latter case, there was little damage to soybean fields despite the high occurrence of male adults near the ground. From the discussion so far, the relationship between *S. litura* and the soybean leaf damage should focus on the dispersal of adults, which can be captured by the searchlight trap. 

Previous studies on *S. litura* pheromone traps corroborate the complex observations discussed above. The male catch numbers of pheromone traps increase almost simultaneously with increases in egg mass numbers on leaves of taro [2,4] and groundnut [5]; however, other catch peaks also occur before or after the increases in egg mass number. For soybean, the moving averages of the male catch of pheromone traps and leaf damage exhibit similar trends, with both peaks occurring almost simultaneously within ±3 days [6]. Thus, an increase in male catch three days before leaf damage suggests the insects’ arrival, which is concordant with the present results. However, the reason for the delay between *S. litura* arrival and soybean leaf damage is unclear. Such delays have also been reported in taro [3]. In general, the variation of pheromone trap catches should be due to two different components induced by separate local and dispersal populations [6]. Because each factor changes independently, it is difficult to clarify the total variation.

In contrast, searchlight traps are sensitive enough to capture dispersal populations, because they capture more insects flying at higher altitudes. In fact, previous studies show that searchlight traps catch more migratory insects than conventional light traps (i.e., 20-W blacklight traps) [9,11,14,24]. Furthermore, in one study conducted in Henan, China, the catch peaks of *Mythimna separata* caught by these 2 traps occurred on different dates, suggesting that the trapped insects were close to or far from the trap locations [24]. Therefore, these findings collectively suggest that searchlight traps are good for capturing both female and male adults of dispersal populations flying at higher altitudes.

Regarding sex ratio, in one study conducted on an island in the Bohai Sea in China from July to October, the average monthly ratio of female *S. litura* caught by a searchlight trap usually exceeded 0.5 [20]. However, in the present study, the ratio of females in large catch peaks in 2016 and 2018 was usually less than 0.5 (Figure 8). Although the reason for this discrepancy is unknown, it should be noted that the flight distance of *S. litura* before being trapped was longer in the previous study than that in the dispersal case in the present study, in which the searchlight trap’s light was turned off at midnight. Therefore, such variations in flight and monitoring conditions might have affected the female ratio of the trap catches.

The results of the present study indicate that pheromone traps are still an effective, inexpensive, and easy-to-use tool for determining the baseline of *S. litura* occurrence. Japanese plant protection stations routinely report total catch numbers of pheromone traps (or average catch number among several traps) every five days for farmers, along with recent maximum and average trends over recent years. However, these catch trends are complex as discussed above, which often make interpretation difficult. Therefore, trap catches alone are insufficient for determining the necessity and timing of chemical pest control. On the other hand, the catches of searchlight traps, which are sensitive to moths flying at higher altitudes, show relatively simpler trends that indicate insect arrival and approximate intensity. However, a major drawback is high costs due to the requisite large trapping equipment, electric power supply, and tedious sample handling, which prevent farmers and farming companies from using this technology. However, searchlight traps can be used by plant protection stations like those in China, because their catch trend data provide useful information about both the regional arrival and intensity of insect pests.

The results of the study are preliminary due to the absence of point replication. Further studies are required for the development in this approach. First, searchlight trap monitoring at multiple sites in the Chikushi Plain is necessary to clarify regional *S. litura* arrival, as well as the optimal number of traps and their configuration for regional pest management. Furthermore, the survival rate of larvae varies with respect to weather. The rate of young instars especially drops during rainy summer days. Linking the trap catches to the economic injury level in the region must also be addressed.

Another important consideration is the timing between *S. litura* arrival and pest control application. Chemical control should be applied during the young larval stage. On the basis of the developmental parameters of *S. litura* [22], the early larval stage lasts for approximately 7 days after hatching at a daily average temperature of 25 °C. Therefore, the time window before pest control application is approximately 10 days after insect arrival. Ten days should be sufficient for plant protection officers and farmers to make forecasts and apply pest control. Accordingly, future work should aim to develop a feasible operational management protocol.

Monitoring long-distance migration of migratory insects must also be considered. Searchlight traps can also catch various migratory insect species besides *S. litura*, such as Noctuidae including *Spodoptera exigua* (Hübner), *Helicoverpa armigera* (Hübner), *M. separata* (Walker), *Agrotis ipsilon* (Hüfnagel), *Ctenoplusia agnata* (Staudinger), Crambidae including *Loxostege sticticalis* (L.), Plutellidae including *Cnaphalocrocis medinalis* (Guenée) and *Plutella xylostella* (L.), and Delphacidae including *Nilaparvata lugens* (Stål) [9,10,11,12,13,14,15,16,17,18,19]. Accordingly, many operational searchlight traps have been set up to monitor such pests in China [25]. An advantage of using searchlight traps in China is that catch numbers are very large. For example, hundreds or even thousands of *M. separata*, *H. armigera*, *S. exigua*, and *S. litura* are frequently caught in one night, especially in autumn [9,11,12,20]. These intense migratory phenomena are probably due to the broad insect source areas around the traps, high insect density, and the relative proximity between the monitoring locations and sources. On the other hand, in the case of overseas migrations, such as those of *S. litura* and *M. separata* traveling between China and Japan, migration paths are longer and hence fewer insects arrive [8,26]. Nevertheless, in order to improve insect monitoring methods in Japan, the effectiveness of monitoring the overseas migration of various insects with searchlight traps must be confirmed and compared with conventional trapping methods.

## Figures and Tables

**Table 1:** Linear regression analysis of trap catch and the number of newly damaged leaves with various date shifts.

Trap Type	Year	Linear Regression Function (X: Trap Catch with Date Shift, Y: Number of Damaged Leaves/1000 Plants) R^2^			
		+3 Days (Date Shift)	+4 Days	+5 Days	+6 Days
Searchlight, Total catch	2016	y = 0.0644x + 6.948 0.1429	y = 0.2387x + 3.7174 0.6118	y = 0.1382x + 6.9351 0.6706 *	y = 0.1168x + 6.4078 0.3609
Searchlight, Total catch	2017	y = 0.0231x + 1.5872 0.0074	y = −0.0726x + 2.2934 0.1401	y = −0.0625x + 2.2698 0.0526 *	y = −0.019x + 1.9376 0.0047
Searchlight, Total catch	2018	y = 0.1676x + 7.5215 0.364	y = 0.2214x + 6.58 0.6143	y = 0.2677x + 5.5721 0.8324	y = 0.2796x + 5.8621 0.8643 *
Searchlight, Female catch	2016	y = 0.1949x + 7.5323 0.1973	y = 0.8029x + 5.2628 0.4018	y = 0.2897x + 8.3447 0.6019 *	y = 0.7056x + 6.2131 0.3869
Searchlight, Female catch	2017	y = 0.0151x + 1.7516 0.0015	y = −0.091x + 2.0366 0.0982	y = −0.0288x + 1.8636 0.0052 *	y = −0.0445x + 1.8925 0.0092
Searchlight, Female catch	2018	y = 0.4775x + 7.6454 0.3273	y = 0.4628x + 9.2734 0.3362	y = 0.7917x + 6.5481 0.6577	y = 0.7809x + 6.7247 0.8403 *
Pheromone, Male catch	2016	y = 0.0155x + 5.0948 0.3027	y = 0.0218x + 4.2323 0.5817	y = 0.0232x + 3.6393 0.6836 *	y = 0.014x + 5.8466 0.312
Pheromone, Male catch	2017	y = −0.0001x + 1.8389 0.0004	y = −0.0031x + 2.6908 0.0536	y = −0.0015x + 2.2395 0.0492 *	y = 0.0014x + 1.5359 0.0077
Pheromone, Male catch	2018	y = 0.0387x − 3.4351 0.6656	y = 0.0459x − 6.9654 0.7432	y = 0.038x − 2.8201 0.7509 *	y = 0.0428x − 4.291 0.7318

* Scatter plots of these functions are shown in Figure 5 and Figure 6.

**Table 2:** Linear regression analysis of cumulative trap catch and cumulative number of damaged leaves with various date shifts.

Trap Type	Year	Linear Regression Function (X: Trap Catch with Date Shift, Y: Number of Damaged Leaves/1000 Plants) R^2^			
		+3 Days (Date Shift)	+4 Days	+5 Days	+6 Days
Searchlight, Cumulative total catch	2016	y = 0.1707x + 12.954 0.9679	y = 0.1764x + 15.309 0.9659	y = 0.1836x + 16.051 0.9749 *	y = 0.1932x + 17.159 0.9702
Searchlight, Cumulative total catch	2017	y = 0.116x + 1.5084 0.9203	y = 0.1195x + 2.1556 0.9090	y = 0.1305x + 1.6895 0.9227	y = 0.1369x + 1.9703 0.9262 *
Searchlight, Cumulative total catch	2018	y = 0.1418x + 29.151 0.9299	y = 0.1483x + 33.794 0.9436 *	y = 0.1584x + 35.617 0.9412	y = 0.1656x + 40.833 0.9374
Searchlight, Cumulative female catch	2016	y = 0.6512x + 34.871 0.8347	y = 0.6852x + 38.035 0.8389	y = 0.745x + 37.437 0.8517	y = 0.8385x + 39.219 0.8571 *
Searchlight, Cumulative female catch	2017	y = 0.3252x + 7.1547 0.6847	y = 0.3529x + 7.4747 0.6604	y = 0.4768x + 5.6777 0.7416	y = 0.6388x + 3.333 0.8195 *
Searchlight, Cumulative female catch	2018	y = 0.4304x + 26.512 0.9171	y = 0.4569x + 30.275 0.9405	y = 0.485x + 32.187 0.9492	y = 0.501x + 37.302 0.9502 *
Pheromone, Cumulative male catch	2016	y = 0.0162x + 26.577 0.9131	y = 0.0168x + 29.57 0.9205 *	y = 0.0181x + 29.455 0.9251	y = 0.0189x + 32.47 0.9335
Pheromone, Cumulative male catch	2017	y = 0.0028x + 0.5951 0.9605 *	y = 0.003x + 0.7741 0.9483	y = 0.003x + 1.247 0.9506	y = 0.0031x + 1.8035 0.9373
Pheromone, Cumulative male catch	2018	y = 0.0129x − 21.695 0.9612 *	y = 0.0136x − 22.553 0.9586	y = 0.014x − 19.519 0.9513	y = 0.0146x − 19.1 0.9312

Date shift (days): The trap catch was shifted by the date shift relative to the number of damaged leaves. * Largest *R*^2^-value in that year.
